# Continued Structural Exploration of Sulfocoumarin as Selective Inhibitor of Tumor-Associated Human Carbonic Anhydrases IX and XII

**DOI:** 10.3390/molecules27134076

**Published:** 2022-06-24

**Authors:** Simone Giovannuzzi, Clemente Capasso, Alessio Nocentini, Claudiu T. Supuran

**Affiliations:** 1 Department of NEUROFARBA, Section of Pharmaceutical and Nutraceutical Sciences, University of Florence, Polo Scientifico, Via U. Schiff 6, Sesto Fiorentino, 50019 Firenze, Italy; simone.giovannuzzi@unifi.it; 2 Istituto di Bioscienze e Biorisorse, CNR, Via Pietro Castellino 111, 80131 Napoli, Italy; clemente.capasso@ibbr.cnr.it

**Keywords:** carbonic anhydrase, cancer, sulfocoumarin

## Abstract

A series of new 3- and 7-substituted sulfocoumarins was obtained by several cyclization reactions and subsequent derivatization for screening as prodrug inhibitors of the human (h) cancer-associated carbonic anhydrases (CAs) IX and XII. All products were ineffective inhibitors against the off-target hCA I and II, whilst hCAs IX and XII were inhibited with inhibition constants (K_I_s) spanning from low nanomolar to the high micromolar range, according to the sulfocoumarin derivatization pattern. In particular, sulfocoumarin 15 turned out to be the most potent and selective inhibitor herein reported (hCA I and II: K_I_ > 100 µM; hCA IX: K_I_ = 22.9 nM; hCA XII: K_I_ = 19.2 nM). Considering that hCA IX and XII validated anti-tumor targets, such prodrug, isoform-selective inhibitors as the sulfocoumarins reported here may be useful for identifying suitable drug candidates for clinical trials.

## 1. Introduction

**Figure 1:**
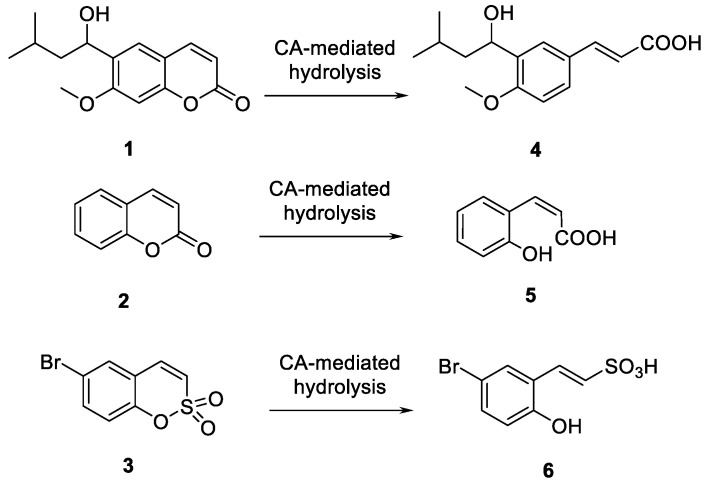
Coumarins **1** and **2,** and sulfocoumarin **3** and their CA-mediated hydrolysis to the CAI active species **4**, **5** and **6**, respectively.

Carbonic anhydrases are a ubiquitous metalloenzyme family present in all living organisms. Until now, eight genetically distinct CA subfamilies have been identified [1,2,3]. In mammals, 16 CA isoforms belonging to α-CAs are characterized, and in humans 15 CA (hCA) isoforms are expressed, showing differences in kinetic properties, subcellular localization and tissue distributions [4,5]. Twelve such isoforms are catalytically active (CAs I–IV, VA, VB, VI, VII, IX, XII and XIV), whereas the remaining three isoforms (VIII, X, XI), called CA-related proteins (CARPs), have no activity. hCAs can be further categorized into four different subsets depending on their subcellular localization as cytosolic (hCA I, II, III, VII, VIII, X, XI, XIII), mitochondrial (hCA VA and VB), secreted (hCA VI) and membrane-bound (hCA IV, IX, XII, and XIV) [5,6,7]. Four distinct CA inhibition mechanisms have been reported and detailed to date with both kinetic and X-crystallographic studies [8,9]. They include (a) the metal ion binders (anions, sulfonamides and their bioisosteres, dithiocarbamates, xanthates, etc.); (b) compounds that anchor to the zinc-coordinated water molecule/hydroxide ion (phenols, carboxylates, polyamines); (c) compounds occluding the active site entrance, such as coumarins and their isosteres (sulfocoumarins); and (d) compounds binding out of the active site, such as an aromatic carboxylic acid derivative [8,9,10]. Coumarins, such as **1** (natural product isolated from the Australian plant Leionema ellipticum) and **2** (the simple unsubstituted coumarin), were discovered as a new chemotype that can inhibit the metalloenzyme carbonic anhydrase a decade ago [11,12,13]. Many differently substituted coumarins were subsequently screened for their inhibitory activity against all the 13 catalytically active mammalian CA isoforms, CA I-VII, IX, XII-XV [14,15,16,17,18]. Many of these isoforms are established drug targets for designing agents with various applications, such as diuretics, antiglaucoma drugs, anticonvulsants, antiobesity agents or antitumor drugs/cancer diagnostic tools [17,18]. To explain the inhibitor mechanism of coumarins **1** and **2**, they were cocrystallized with human CA II, and the electron density data showed the presence of the hydrolyzed derivatives **4** and **5**, respectively (Figure 1). The most notable aspect of this inhibition mechanism is the fact that the **4** and **5** occlude the enzyme active site binding at the entrance of the cavity.

**Figure 2:**
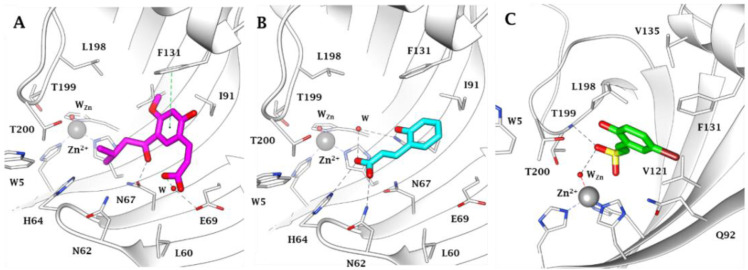
A ribbon view of the active site of hCA II in adduct with (**A**) Compound **4** (cyan, PDB 5BNL), derived from the CA-mediated hydrolysis of coumarin **1**; (**B**) Compound **5** (magenta, PDB 3F8E), derived from the CA-mediated hydrolysis of coumarin **2**. (**C**) Compound **6** (green, PDB 4BCW), derived from the CA-mediated hydrolysis of sulfocoumarin **3**. The Zn(II) is shown as a grey sphere that is bound to the protein His ligands (labels not shown). Water molecules are represented as red spheres. H-bonds are represented as black dashed lines.

Over the last several years, a rather large number of new classes of CAIs were reported, starting from coumarins as the lead, among which are thiocoumarins, sulfocoumarins, 2-thioxo-coumarins, coumarin oximes, 5-/6-membered/thio)lactones, etc. [19,20,21,22,23,24,25,26,27,28]; coumarins and sulfocoumarins were also shown to be isoform-selective CA inhibitors. Particularly, in 2012, the sulfocoumarins were identified as CAIs using kinetic and X-ray crystallographic studies, in which it has been also revealed that although structurally related to the coumarins, sulfocoumarins possess a different CA inhibition mechanism (Figure 2) [29]. As reported in Figure 1 for compound **3**, sulfocoumarin were hydrolyzed by α-CAs to give trans-2-hydroxyphenyl-ω-ethenylsulfonic acid. Afterwards, the formed sulfonic acid binds to the CA II active site by anchoring the SO_3_H group to the zinc-coordinated water molecule/hydroxide ion (Figure 2) [30]. After this discovery, many more derivatives were synthesized and analyzed for their interaction with different CA isoforms [31,32,33,34,35,36,37].

The substitution pattern, and especially the position of the substituent on the heterocyclic ring system of the sulfocoumarin, are the main factors influencing CA inhibitory properties [30]. In this paper, we expanded the structure-activity relationships of the sulfocoumarin scaffold describing the synthesis and the evaluation of more than 30 sulfocoumarin belonging to two different classes: (a) 7-benzyloxysulfocoumarin and (b) 3-amidosulfocoumarin, obtained for the first time in this work.

## 2. Results

### 2.1. Chemistry

**Figure 3:**
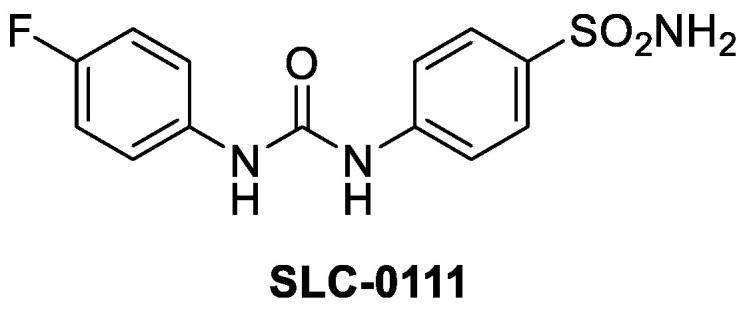
The structure of SLC-0111, a specific hCA IX and XII inhibitor studied in phase Ib/II clinical trials for the treatment of hypoxic tumors.

Due to the difficulties in designing and synthesizing selective sulfonamide inhibitors against each isoform, such as SLC-0111 (Figure 3) [38], a potent and selective zinc binder CAI against hCA IX and XII, scientists opted for the development of novel chemotypes among which are sulfocoumarins, the preferred CAI scaffold adopted in this project.

**Scheme 1:**
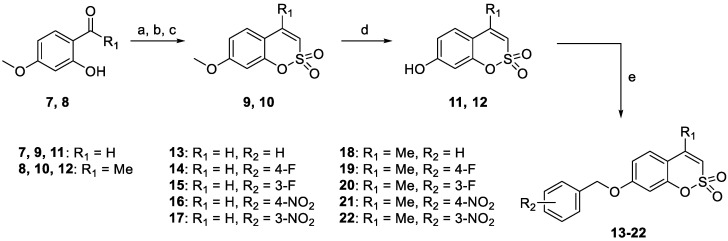
A synthetic approach for obtaining products **13**–**22**. Reagents and conditions: (a) Mesylchloride, Et_3_N, dry DCM, 0 °C to RT, 2 h; (b) DBU, dry DCM, 0 °C to RT, o.n.; (c) dry Py, dry DCM, 0 °C to RT, o.n.; (d) BBr_3_, dry DCM, *−*10 °C to RT, o.n.; (e) appropriate benzyl bromide, K_2_CO_3_, dry DMF, RT, o.n.

The general strategy of Zalubovskis group [16,29], for the preparation of 6-substituted sulfocoumarins and validated by Nocentini et al. [36], in 2015, for the synthesis of 7-substituted such derivatives, was applied in this manuscript to extend the structure-activity relationships, whereas the general strategy of Liu’s group [39], for the designed of 3-substituted sulfocoumarins, was validated in this project in order to synthesize 3-amido derivatives for the first time. To start, 2-hydroxy-4-methoxybenzaldehyde **7** or 2′-hydroxy-4′-methoxyacetophenon **8** were synthesized with the sulfocoumarin scaffold **9** and **10**. After that, their phenol moiety was released in the presence of BBr_3_ in dry DCM to obtain **11** and **12** in high yield and high purity. Finally, a nucleophilic substitution was performed in the presence of different benzyl bromides with K_2_CO_3_ as a base in dry DMF at RT to give compounds **13**–**22** (Scheme 1).

**Scheme 2:**
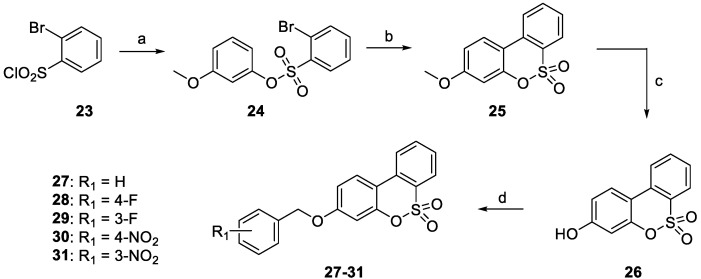
A synthetic approach to obtain products **27**–**31**. Reagents and conditions: (a) 3-methoxyphenol, Et_3_N, dry DCM, 0 °C to RT, 3 h; (b) KOAc, Pd(OAc)_2_, dry DMA, 150 °C, 4 h; (c) BBr_3_, dry DCM, *−*10 °C to RT, o.n.; (d) appropriate benzyl bromide, K_2_CO_3_, dry DMF, RT, o.n.

Compound **23** (2-bromobenzenesulfonyl chloride) was reacted with 3-methoxyphenol in dry DCM in the presence of Et_3_N as a base to obtain intermediate **24**; after that, a cyclization was performed in dry DMA with Pd(OAc)_2_ as a catalyst at 150 °C to give the sulfocoumarin **25**. To prepare the next reaction, the methoxy group was released in the presence of BBr3 in dry DCM between *−*10 °C and RT to synthesize intermediate **26,** and then a nucleophilic substitution was performed in dry DMF at RT with K_2_CO_3_ as a base to give us compounds **27**–**31** (Scheme 2).

**Scheme 3:**
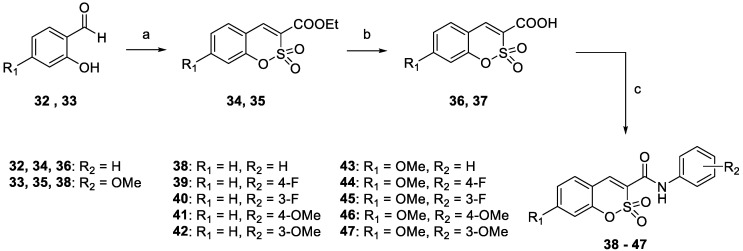
A synthetic approach to obtain products 38–47. Reagents and conditions: (a) ethyl 2-chlorosulfonyl acetate, dry Py, DCE, 90 °C, 2 h; (b) NaOH 5M, EtOH, reflux, 30′; (c) appropriate aniline, PyBOP, DIPEA, dry DMF, 0 °C to RT, o.n.

A cyclization reaction was performed between starting materials **32**, or salicylaldehyde, or **33**, 4-methoxysalicylaldehyde, and ethyl 2-chlorosulfonylacetate in DCE at 90 °C in the presence of dry pyridine as a base; after that, the obtained ethyl esters (**34**, **35**) were hydrolyzed in EtOH and NaOH 5M at reflux to release the carboxylic acid moiety of compounds **36** and **37**. Finally, a coupling reaction was performed in dry DMF between appropriate anilines and intermediates **36** and **37** to give us products **38**–**47** with an amide as linker (Scheme 3).

### 2.2. Carbonic Anhydrase Inhibition

Sulfocoumarins **13**–**22**, **27**–**31**, **38**–**47** were screened in vitro for the inhibition of four physiologically relevant hCA isoforms, the cytosolic hCAI and II and the trans-membrane tumor-associated hCA IX and XII [4,5,6,8,40,41,42,43]; acetazolamide (AAZ) was used as standard CAI. CA I is the main off-target isoform for most therapeutic applications of CAIs, whilst CA II is considered off-target in many pathologies to reduce side effects resulting from systematic CA inhibition as much as possible. Table 1 shows the inhibition data obtained after a period of incubation of 6 h of the enzyme and inhibitors. Noteworthily, the assay inhibition performed within the usual 15 min incubation period (as for the sulfonamides) led to the very weak inhibition constants (data not shown) [43]. For this reason, we herein report a 6 h incubation time instead. The following structure-activity relationship (SAR) can be gathered from the inhibition data reported in Table 1.

The following structure-activity relationships (SAR) should be noted:

- According to the previous reports [31,32,33,35], isoform hCA I was not inhibited by a large number of substituted sulfocoumarins; however, on the other side, the simplest sulfocoumarin as **11**, **12**, **26**, **36** and **37** showed a weak inhibitory potency. It is likely that compounds **36** (K_I_ = 4625 nM) and **37** (K_I_ = 7139 nM) have a low micromolar inhibition due to the presence of the carboxylic acid moiety, which may work by binding to the H_2_O molecule coordinated with Zn^2+^ in the active site [45,46].
- Similar to hCA I, for hCA II we did not observe any inhibition by all the substituted sulfocoumarines, except for **9**–**12**, **26**, **36** and **37**. Compounds **11** and **12** showed a low micromolar inhibition, respectively K_I_ = 2896 nM and K_I_ = 3857 nM, whilst analogs **9** and **10** have the K_I_ in the medium micromolar range, which can likely be associated to the presence of a free phenol moiety on products **11** and **12**, which is protected in **9** and **10**.
- The target hCA IX resulted in being the most inhibited isoforms by sulfocoumarins reported here. Considering the three different types of products, type 1 shows the most efficient inhibition against this isozyme, with K_I_ values in the low-medium nanomolar range, between 14.3 nM and 97.2 nM. In detail, the first-in-class compound in term of inhibition potency is the number **19**, which shows a fluorine atom in para position on the benzyloxy moiety and a methyl group as R1 substituent (K_I_ = 14.3 nM), whilst the elimination of the benzyloxy moiety (**10**) gives us the less effective compound (K_I_ = 97.2 nM). Interestingly, the general behaviour of these compounds owned on type 1 was that, indeed, the insertion of the methyl group as an R1 substituent produced a worsening of the K_I_ values; in fact, it was demonstrated by products **16** (K_I_ = 68.9 nM) and **17** (K_I_ = 40.2 nM) compared to the corresponding **21** (K_I_ = 89.7 nM) and **22** (K_I_ = 61.4 nM). This rule seems to be broken only by derivatives **18** (K_I_ = 49.8 nM) and **19** (K_I_ = 14.3 nM), which resulted in better inhibitors with respect to analogs **13** (K_I_ = 55.6 nM) and **14** (K_I_ = 22.9 nM). Sulfocoumarines belonging to type 2, even with a benzyloxy moiety, suffered from the addition of a further aromatic ring on the scaffold that improves the steric hindrance on the sulfonate ring, which induced a worsening of the K_I_ values. Indeed, the inhibition constants are in the high nanomolar range, between 615.1 nM and 1286 nM. The best compound result to be **29** (K_I_ = 615.1 nM) with a fluorine atom in meta position on the benzyloxy moiety. The movement of the R1 group from meta to para position produces a worsening of the K_I_ values. Examples are **29** (K_I_ = 615.1 nM) and **31** (K_I_ = 898.1 nM), respectively, with 28 (K_I_ = 754.9 nM) and **30** (K_I_ = 964.7 nM). Interestingly, it is a significant decline of the inhibition data of the sulfocoumarines belonging to type 3, with an amide as linker on position 3 of the sulfonate ring. It is likely that the presence of bulky groups with the CAI portion results in a worsening of the K_I_ values, in a micromolar range between 499.7 nM and 64380 nM. The most potent compounds of this class are **43** (K_I_ = 2429 nM) and the simplest sulfocoumarines **34** (K_I_ = 1631 nM), **35** (K_I_ = 1085 nM), **36** (K_I_ = 499.7 nM) and **37** (K_I_ = 853.7 nM) showed an ester or carboxylic acid moieties in position 3. Sulfocoumarines with a bromine atom in a meta position as the R2 group (**40**, **45**) resulted in the worst values of inhibition, respectively K_I_ = 40,840 nM and 64,380 nM. The hCA XII resulted in becoming the second most efficiently inhibited isoform by sulfocoumarines, with it being particularly possible to observe a similar trend of the K_I_ values as already shown for the hCA IX. Considering products belonging to type 1, they show the most potent inhibition for this isoform, between 8.6 nM and 136.0 nM. The presence of a methyl group in R1 position produced an improvement of the K_I_ values for derivatives **18** (K_I_ = 8.6 nM), without substituent on the benzyloxy moiety, and **20** (K_I_ = 25.7 nM), with fluorine in the meta position. For all the other products, a worsening of Ki values, such as for **14** (K_I_ = 19.2 nM) and **19** (K_I_ = 41.7 nM), or for **11** (K_I_ = 42.5 nM) and **12** (K_I_ = 55.0 nM), was observed. On the other side, the movement of the fluorine atom from meta to para position (**19**) resulted in a K_I_ value of 41.7 nM. Regarding the nitro group, compound **17** (K_I_ = 77.5 nM) with R2 group in a meta position show an improvement in term of inhibition than its analogue **16** (K_I_ = 94.7 nM) with R2 group in a para position. For sulfocoumarines **18**–**22**, the fluorine atom resulted in being better than nitro group as a substituent on the benzyloxy moiety; indeed, **19** (K_I_ = 41.7 nM) and **20** (K_I_ = 25.7 nM) are strongest inhibitors in term of potency than **21** (K_I_ = 100.8 nM) and **22** (K_I_ = 136.0 nM). For compounds belonging to type 2, the addition of an aromatic ring on the sulfocoumarin scaffold determines a worsening of the K_I_ values in the medium–high nanomolar range, between 414.2 nM and 1369 nM. The presence of a bulky group such as the nitro group leads to a worsening in terms of inhibition potency, particularly products **30** and **31** have K_I_ of 1056 nM and 1369 nM. The simplest sulfocoumarin with a free phenol moiety, **26**, showed the best inhibition for type 2 series, 414.2 nM. Sulfocoumarins belonging to type 3 that present a further enhancement of the steric hindrance on the sulfonate ring, have K_I_ values in the range of micromolar (K_I_ = 408.2–78,320 nM). Compound **40** and **45**, with a bromine atom in the meta position, show the worst inhibition potencies on hCA XII, respectively 78,320 nM and 76,540 nM; on the other side, sulfocoumarines with the less bulky group in R_2_ position result in being a good inhibitor on this isoform, while, indeed, **38** and **43** with a hydrogen atom show K_I_ values in the range of low micromolar—respectively, 2301 nM and 1594 nM. The best inhibitors belonging to the type 3 series are compounds **36** (K_I_ = 563.7 nM) and **37** (K_I_ = 408.2 nM), likely thanks to the free carbocylic acid moiety.

## 3. Materials and Methods

### 3.1. Chemistry

Anhydrous solvents and all reagents were purchased from Merck, Fluorochem and TCI. All reactions involving air- or moisture-sensitive compounds were performed under a nitrogen atmosphere using dried glassware and syringes techniques to transfer solutions. Nuclear magnetic resonance (1H-NMR, 13C-NMR,) spectra were recorded using a Bruker Advance III 400 MHz spectrometer in DMSO-d_6_. Chemical shifts are reported in parts per million (ppm), and the coupling constants (J) are expressed in Hertz (Hz). Splitting patterns are designated as follows: s, singlet; d, doublet; t, triplet; q, quadruplet; m, multiplet; bs, broad singlet; dd, doublet of doublets. The assignment of exchangeable protons was confirmed by the addition of D_2_O. Analytical thin-layer chromatography (TLC) was carried out on Sigma Aldrich silica gel F-254 plates. Flash chromatography purifications were performed on Sigma Aldrich Silica gel 60 (230–400 mesh ASTM) as the stationary phase and ethyl acetate/n-hexane or MeOH/DCM were used as eluents. Melting points (mp) were measured in open capillary tubes with a Gallenkamp MPD350.BM3.5 appa-ratus and are uncorrected. The solvents used in mass spectrometry analysis were acetone, acetonitrile (Chromasolv grade), purchased from Sigma-Aldrich (Milan, Italy), and mQ water 18 MΩ cm, obtained from Millipore’s Simplicity system (Milan, Italy). The HPLC-MS and MS/MS analysis was carried out using a Varian 500-MS ion trap system (Palo Alto, CA, USA) equipped by two Prostar 210 pumps, a Prostar 410 autosampler and an Electrospray source (ESI) operating in negative ions. Stock solutions of analytes were prepared in acetone at 1.0 mg mL-1 and stored at 4 °C. Working solutions of each analyte were freshly prepared by diluting stock solutions in a mixture of mQ water:acetonitrile 1:1 (*v*/*v*) up to a concentration of 1.0 µg mL^−1^. The mass spectra of each analyte were acquired by introducing, via syringe pump at 10 µL min^−1^, the working solution. Raw data were collected and processed by Varian Workstation Vers. 6.8 software.

**General synthetic procedure for 7-methoxybenzo[e][1,2]oxathiine 2,2-dioxide (9) and 7-methoxy-4-methylbenzo[e][1,2]oxathiine 2,2-dioxide (10)**

Et_3_N (1.5 equiv.) and Mesyl chloride (1.5 equiv.) were added slowly to a solution of **7** or **8** (3 g, 1 equiv.) in dry DCM (20 mL) at 0 °C under nitrogen atmosphere. The solution was stirred for 2 h at RT. Slush was added to the mixture and the reaction mixture was extracted in DCM (3 × 25 mL), dried with Na_2_SO_4_, filtered and evaporated to give us a pale yellow oil. DBU (1.2 equiv.) was added dropwise to a solution of the oil in dry DCM (20 mL) at 0 °C under nitrogen atmosphere. The solution was stirred o.n. at RT. Slush was added slowly and the reaction mixture was extracted in DCM (3 × 25 mL). The collected organic phases were dried with Na_2_SO_4_, filtered and evaporated under vacuum to give a brown oil. POCl_3_ (1.5 equiv.) was added dropwise to a solution of the oil in dry Py (3 mL) at 0 °C under nitrogen atmosphere. The solution was stirred on at RT. Ice was added slowly to the reaction mixture and the resulting precipitate was filtered to give intermediates **9** and **10** as a light brown powder in high yield. **9** and **10** were purified by silica gel chromathography column (EtOAc/Hexane: from 20% to 60% *v*/*v*) to give white-grey powder.

***7-Methoxybenzo[e][1,2]oxathiine 2,2-dioxide (*9*)***

Compound **9** was obtained according to the general procedure reported earlier with 4-methoxysalicyl aldehyde (**7**) as starting material. Yield 81%; m.p. 111–112 °C; silica gel TLC Rf 0.82 (MeOH/DCM 5% *v*/*v*); δ_H_ (400 MHz, DMSO-d_6_): 7.65 (d, *J* = 10.0 Hz, 1H, Ar-*H*), 7.63 (d, *J* = 8.4 Hz, 1H, Ar-*H*), 7.32 (d, *J* = 10.0 Hz, 1H, Ar-*H*), 7.09 (d, *J* = 2.2 Hz, 1H, Ar-*H*), 7.01 (dd, *J* = 8.4 2.2 Hz, 1H, Ar-*H*), 3.88 (s, 3H, C*H*_3_); δ_C_ (400 MHz, DMSO-d_6_): 163.3, 153.3, 137.4, 132.0, 120.0, 113.8 112.8, 104.7, 57.0.

***7-Methoxy-4-methylbenzo[e][1,2]oxathiine 2,2-dioxide (*10*)***

Compound **10** was obtained according to the general procedure reported earlier with 2-hydroxy-4-methoxyacetophenon (**8**) as starting material. Yield 76%; m.p. 151–153 °C; silica gel TLC Rf 0.79 (MeOH/DCM 5% *v*/*v*): 7.73 (d, *J* = 8.2 Hz, 1H, Ar-*H*), 7.24 (s, 1H, Ar-*H*), 7.10 (s, 1H, Ar-*H*), 7.05 (d, *J* = 8.2 Hz, 1H, Ar-*H*), 3.90 (s, 3H, CH_3_), 2.37 (s, 3H, C*H*_3_); δ_H_ (400 MHz, DMSO-d_6_); δ_C_ (400 MHz, DMSO-d_6_): 157.5, 154.3, 142.7, 126.8, 119.3, 107.6, 107.3, 106.8, 558, 19.9.

**Synthetic procedure for 3-methoxyphenyl 2-bromobenzenesulfonate (24)**

Et_3_N (2 equiv.) and 2-bromobenzenesulfonyl chloride (**23**) (1 equiv.) were added slowly to a solution of 3-methoxyphenol (0.5 g, 1.1 equiv.) in dry DCM at 0 °C under nitrogen atmosphere. The solution was stirred for 4 h at RT. Slush and HCl 2M were added to pH = 4 and the reaction mixture was extracted in DCM (3 × 15 mL). The collected organic phases were washed with K_2_CO_3_ s.s. (2 × 10 mL), dried with Na_2_SO_4_, filtered and evaporated under vacuum to give products **24** as white powder in high yield and purity.

Yield 96%; m.p. 163–166 °C; silica gel TLC Rf 0.45 (EtOAc/Hexane 50% *v*/*v*); δ_H_ (400 MHz, DMSO-d_6_): 8.08 (d, *J* = 8.4 Hz, 1H, Ar-*H*), 7.99 (d, *J* = 8.4 Hz, 1H, Ar-*H*), 7.75 (dd, *J* = 7.4 Hz, 1H, Ar-*H*), 7.65 (dd, *J* = 7.4 Hz, 1H, Ar-*H*), 7.33 (dd, *J* = 7.1 Hz, 1H, Ar-*H*), 6.93 (d, *J* = 7.1 Hz, 1H, Ar-*H*), 6.70 (m, 2H, Ar-*H*), 3.73 (s, 3H, C*H*_3_); δ_C_ (400 MHz, DMSO-d_6_): 162.0, 152.7, 146.8, 134.7, 131.7, 134.7, 131.1, 130.3, 129.0, 115.6, 106.9, 102.0, 55.8.

**Synthetic procedure for 3-methoxydibenzo[c,e][1,2]oxathiine 6,6-dioxide (25)**

Potassium acetate (2 equiv.) and Pd(OAc)_2_ (0.01 equiv.) were added to a solution of **24** (1.2 g, 1 equiv.) in dry DMA (5 mL) under nitrogen atmosphere. The solution was heated at 150 °C for 4 h. The Resulting mixture was cooled in ice bath and slush was added. The suspension was extracted in EtOAc (3 × 20 mL), and the collected organic phases were washed with brine (3 × 50 mL), dried with Na_2_SO_4_, filtered and evaporated under vacuum to give **25** as red oil. Product was purified by silica gel chromathography column (MeOH/DCM: from 0.5% to 1% *v*/*v*) to obtain a white powder.

Yield 93%; m.p. 180–183 °C; silica gel TLC Rf 0.45 (EtOAc/Hexane 50% *v*/*v*); δ_H_ (400 MHz, DMSO-d_6_): 8.25 (dd, J = 8.3 Hz, 2H, Ar-H), 8.07 (dd, J = 8.3 Hz, 2H, Ar-H), 7.95 (m, 1H, Ar-H), 7.72 (m, 1H, Ar-H), 7.20 (s, 1H, Ar-H), 7.15 (dd, J = 8.3 Hz, 1H, Ar-H), 3.92 (s, 3H, CH_3_); δ_C_ (400 MHz, DMSO-d_6_): 160.9, 157.2, 142.4, 134.4, 133.0, 130.3, 128.9, 128.6, 122.7, 119.4, 102.5, 55.8.

**General syndhetic procedure for 7-hydroxybenzo[e][1,2]oxathiine 2,2-dioxide (11),7-hydroxy-4-methylbenzo[e][1,2]oxathiine 2,2-dioxide (12) and 3-hydroxydibenzo[c,e][1,2]oxathiine 6,6-dioxide (26)**

BBr_3_ (5 equiv.) was added dropwise and slowly to a solution of **9**, **10** or **25** (2 g, 1 equiv.) in dry DCM (15 mL) at −20 °C under nitrogen atmosphere. The solution was stirred 24 h at RT. Ice was added slowly to the solution and the precipitate was filtered. The resulting powder was dissolved in K_2_CO_3_ s.s. (15 mL) and was filtered off. The H_2_O-phase was acidified with HCl 6 M to pH = 4 and the product was extracted in EtOAc (3 × 20 mL), dried with Na_2_SO_4_, filtered and evaporated under vacuum to give products **11**, **12** and **26** as white powder in good yield and high purity.

***7-Hydroxybenzo[e][1,2]oxathiine 2,2-dioxide (*11*)***

Compound **11** was obtained according to the general procedure reported earlier with **9** as starting material. Yield 72%; m.p. 157–159 °C; silica gel TLC Rf 0.41 (EtOAc/Hexane 50% *v*/*v*); δ_H_ (400 MHz, DMSO-d_6_): 10.81 (s, 1H, exchange with D_2_O, O*H*), 7.59 (d, *J* = 9.8 Hz, 1H, Ar-*H*), 7.52 (d, *J* = 8.9 Hz, 1H, Ar-*H*), 7.22 (d, *J* = 9.8 Hz, 1H, Ar-*H*), 6.82 (dd, *J* = 8.9 1.4 Hz, 1H, Ar-*H*), 6.75 (d, *J* = 1.4 Hz, 1H, Ar-*H*); δ_C_ (400 MHz, DMSO-d_6_): 159.8, 155.5, 133.4, 131.2, 126.7, 109.1, 108.4, 106.5.

***7-Hydroxy-4-methylbenzo[e][1,2]oxathiine 2,2-dioxide (*12*)***

Compound **12** was obtained according to the general procedure reported earlier with **10** as starting material. Yield 70%; m.p. 172–175 °C; silica gel TLC Rf 0.38 (EtOAc/Hexane 50% *v*/*v*); δ_H_ (400 MHz, DMSO-d_6_): 10.84 (s, 1H, exchange with D_2_O, O*H*), 7.63 (d, *J* = 8.7 Hz, 1H, Ar-*H*), 7.14 (s, 1H, Ar-*H*), 6.87 (d, *J* = 8.7 Hz, 1H, Ar-*H*), 6.77 (s, 1H, Ar-*H*), 2.34 (s, 3H, C*H*_3_); δ_C_ (400 MHz, DMSO-d_6_): 159.0, 155.1, 142.7, 127.2, 119.3, 108.4, 107.9, 106.2, 19.9.

***3-Hydroxydibenzo[c,e][1,2]oxathiine 6,6-dioxide (*26*)***

Compound **26** was obtained according to the general procedure reported earlier with **25** as starting material. Yield 86%; m.p. 181–182 °C; silica gel TLC Rf 0.44 (EtOAc/Hexane 50% *v*/*v*); δ_H_ (400 MHz, DMSO-d_6_): 10.68 (s, 1H, exchange with D_2_O, O*H*), 8.19 (d, *J* = 7.9 Hz, 1H, Ar-*H*), 8.12 (d, *J* = 7.9 Hz, 1H, Ar-*H*), 8.0 (d, *J* = 7.4 Hz, 1H, Ar-*H*), 7.92 (dd, *J* = 7.4 Hz, 1H, Ar-*H*), 7.68 (dd, *J* = 7.4 Hz, 1H, Ar-*H*), 6.97 (d, *J* = 7.4 Hz, 1H, Ar-*H*), 6.88 (s, 1H, Ar-*H*); δ_C_ (400 MHz, DMSO-d_6_): 158.8, 157.6, 142.4, 134.4, 134.4, 133.0, 130.7, 128.6, 122.3, 119.8, 109.0, 104.1.

**General synthetic procedure for products 13–22, 27–31**

K_2_CO_3_ (1.05 equiv.) and the appropriate benzyl bromide (0.95 equiv.) were added to a solution of **11**, **12** or **26** (0.15 g, 1 equiv.) in dry DMF (2 mL) under nitrogen atmosphere. The suspension was stirred o.n. at RT. The reaction mixture was quenched with slush and the H_2_O-phase was extracted in EtOAc (3 × 25 mL). The organic phases were collected and were washed with NaOH 5M (3 × 10 mL) and brine (3 × 50 mL), then organic phase was dried with Na_2_SO_4_, filtered and evaporated under vacuum to obtain products as white powder in high yield and purity.

**7-(Benzyloxy)benzo[e][1,2]oxathiine 2,2-dioxide (13)**

Compound **13** was obtained according to the general procedure reported earlier with benzyl bromide as starting material. Yield 74%; m.p. 165–167 °C; silica gel TLC Rf 0.45 (EtOAc/Hexane 50% *v*/*v*); δ_H_ (400 MHz, DMSO-d_6_): 7.65 (d, *J* = 9.1 Hz, 2H, Ar-*H*), 7.41 (m, 6H, Ar-*H*), 7.18 (s, 1H, Ar-*H*), 7.08 (d, *J* = 8.5 Hz, 1H, Ar-*H*), 5.23 (s, 2H, C*H*_2_); δ_C_ (400 MHz, DMSO-d_6_): 162.4, 153.3, 137.5, 137.1, 132.2, 129.6, 129.2, 129.0, 120.3, 114.5, 113.1, 105.5, 71.1; *m*/*z* (ESI negative): 286.9 [M − H]^−^, *m*/*z* (ESI positive): 289.0 [M + H]^+^.

**7-((4-Fluorobenzyl)oxy)benzo[e][1,2]oxathiine 2,2-dioxide (14)**

Compound **14** was obtained according to the general procedure reported earlier with 4-fluorobenzyl bromide as starting material. Yield 77%; m.p. 197–199 °C; silica gel TLC Rf 0.39 (EtOAc/Hexane 50% *v*/*v*); δ_H_ (400 MHz, DMSO-d_6_): 7.68 (s, 1H, Ar-*H*), 7.66 (d, *J* = 2.5 Hz, 1H, Ar-*H*), 7.58 (m, 2H, Ar-*H*), 7.34 (d, *J* = 10.3 Hz, 1H Ar-*H*), 7.28 (dd, *J* = 8.9 Hz, 2H, Ar-*H*), 7.20 (d, *J* = 2.3 Hz, 1H, Ar-*H*),7.10 (dd, *J* = 8.9 2.3 Hz, 1H, Ar-*H*), 5.24 (s, 2H, C*H*_2_); δ_C_ (400 MHz, DMSO-d_6_): 164.2 (d, *J*^1^ = 244.0 Hz), 162.3, 153.3, 137.5, 133.4, 132.2, 131.4 (d, *J*^3^ = 8.4 Hz), 120.3, 116.5 (d, *J*^2^ = 21.5 Hz), 114.5, 113.1, 105.7, 70.4; *m*/*z* (ESI negative): 304.9 [M − H]^−^, *m*/*z* (ESI positive): 307.0 [M + H]^+^.

***7-((3-Fluorobenzyl)oxy)benzo[e][1,2]oxathiine 2,2-dioxide (*15*)***

Compound **15** was obtained according to the general procedure reported earlier with 3-fluorobenzyl bromide as starting material. Yield 76%; m.p. 180–183 °C; silica gel TLC Rf 0.38 (EtOAc/Hexane 50% *v*/*v*); δ_H_ (400 MHz, DMSO-d_6_): 7.68 (m, 2H, Ar-H), 7.51 (m, 2H, Ar-H), 7.36 (m, 3H, Ar-H), 7.21 (s, 1H, Ar-H), 7.12 (d, J = 7.1 Hz, 1H, Ar-H), 5.29 (s, 2H, C*H*_2_); δ_C_ (400 MHz, DMSO-d_6_): 164.4 (d, *J*^1^ = 241.1 Hz), 62.1, 153.3, 140.0 (d, *J*^3^ = 7.3 Hz), 137.5, 132.2, 131.6 (d, *J*^3^ = 7.6 Hz), 124.8 (d, *J*^4^ = 2.4 Hz), 120.4, 116.0 (d, *J*^2^ = 20.4 Hz), 115.6 (d, *J*^2^ = 21.7 Hz), 114.5, 113.3, 105.7, 70.2; *m*/*z* (ESI negative): 304.9 [M − H]^−^, *m*/*z* (ESI positive): 307.0 [M + H]^+^.

***7-((4-Nitrobenzyl)oxy)benzo[e][1,2]oxathiine 2,2-dioxide (*16*)***

Compound **16** was obtained according to the general procedure reported earlier with 4-nitrobenzyl bromide as starting material. Yield 82%; m.p. 215–217 °C; silica gel TLC Rf 0.48 (EtOAc/Hexane 50% *v*/*v*); δ_H_ (400 MHz, DMSO-d_6_): 8.32 (d, *J* = 8.6 Hz, 2H, Ar-*H*), 7.78 (d, *J* = 8.6 Hz, 2H, Ar-*H*), 7.70 (d, *J* = 8.4 Hz, 1H, Ar-*H*), 7.68 (d, *J* = 10.0 Hz, 1H, Ar-*H*), 7.36 (d, *J* = 10.0 Hz, 1H, Ar-*H*), 7.23 (d, *J* = 2.3 Hz, 1H, Ar-*H*), 7.14 (dd, *J* = 8.4 2.3 Hz,1H, Ar-*H*), 5.29 (s, 2H, C*H*_2_); δ_C_ (400 MHz, DMSO-d_6_): 161.9, 153.3, 148.2, 144.9, 137.5, 132.3, 129.5, 124.7, 120.5, 114.5, 113.4, 105.8, 69.8; *m*/*z* (ESI negative): 331.9 [M − H]^−^, *m*/*z* (ESI positive): 334.0 [M + H]^+^.

**7-((3-nitrobenzyl)oxy)benzo[e][1,2]oxathiine 2,2-dioxide** (**17**)

Compound **17** was obtained according to the general procedure reported earlier with 3-nitrobenzyl bromide as starting material. Yield 81%; m.p. 200–202 °C; silica gel TLC Rf 0.51 (EtOAc/Hexane 50% *v*/*v*); δ_H_ (400 MHz, DMSO-d_6_): 8.37 (s, 1H, Ar-H) 8.2 (d, J = 8.1 Hz, 1H, Ar-H), 7.96 (d, J = 8.1 Hz, 1H, Ar-H), 7.71 (m, 3H, Ar-H), 7.34 (d, J = 10.3 Hz, 1H, Ar-H), 7.22 (d, J = 2.8 Hz, 1H, Ar-H), 7.12 (dd, J = 8.3 2.8 Hz, 1H, Ar-H), 5.40 (s, 2H, C*H*_2_); δ_C_ (400 MHz, DMSO-d_6_): 162.0, 153.3, 148.9, 139.5, 137.5, 135.4, 132.3, 131.2, 124.1, 123.4, 120.5, 114.5, 113.4, 105.8, 69.8; *m*/*z* (ESI negative): 332.0 [M − H]^−^, *m*/*z* (ESI positive): 334.0 [M + H]^+^.

**7-(benzyloxy)-4-methylbenzo[e][1,2]oxathiine 2,2-dioxide** (**18**)

Compound **18** was obtained according to the general procedure reported earlier with benzyl bromide as starting material. Yield 83%; m.p. 174–175 °C; silica gel TLC Rf 0.41 (EtOAc/Hexane 50% *v*/*v*); δ_H_ (400 MHz, DMSO-d_6_): 7.73 (d, *J* = 9.0 Hz, 1H, Ar-*H*), 7.51 (d, *J* = 7.1 Hz, 2H, Ar-*H*), 7.45 (dd, *J* = 7.1 Hz, 2H, Ar-*H*), 7.40 (q, *J* = 7.1 Hz, 1H, Ar-*H*), 7.23 (s, 1H, Ar-*H*), 7.19 (d, *J* = 2.1 Hz, 1H, Ar-*H*), 7.12 (dd, *J* = 9.0 2.1 Hz, 1H, Ar-*H*), 5.27 (s, 2H, C*H*_2_), 2.37 (s, 3H, C*H*_3_); δ_C_ (400 MHz, DMSO-d_6_): 162.3, 152.4, 146.6, 137.1, 129.6, 129.3, 129.2, 129.0, 117.3, 114.7, 114.3, 105.7, 71.1, 20.0; *m*/*z* (ESI negative): 300.9 [M − H]^−^, *m*/*z* (ESI positive): 303.0 [M + H]^+^.

**7-((4-fluorobenzyl)oxy)-4-methylbenzo[e][1,2]oxathiine 2,2-dioxide** (**19**)

Compound **19** was obtained according to the general procedure reported earlier with 4-fluorobenzyl bromide as starting material. Yield 73%; m.p. 195–198 °C; silica gel TLC Rf 0.43 (EtOAc/Hexane 50% *v*/*v*); δ_H_ (400 MHz, DMSO-d_6_): 7.74 (d, *J* = 8.9 Hz, 1H, Ar-*H*) 7.57 (m, 2H, Ar-*H*), 7.28 (m, 3h, Ar-*H*), 7.24 (s, 1H, Ar-*H*), 7.19 (d, *J* = 2.1 Hz, 1H, Ar-*H*), 7.12 (dd, *J* = 8.8 2.1 Hz, 1H, Ar-*H*), 5.25 (s, 2H, C*H*_2_), 2.37 (s, 3H, C*H*_3_); δ_C_ (400 MHz, DMSO-d_6_): 164.27 (d, *J*^1^ = 214.7 Hz), 162.2, 152.4, 164.5, 133.4 (d, *J*^4^ = 2.5 Hz), 131.3 (d, *J*^2^ = 8.8 Hz), 129.3, 117.4, 116.5 (d, *J*^2^ = 20.7 Hz), 114.7, 114.3, 105.7, 70.3, 20.0; *m*/*z* (ESI negative): 318.9 [M − H]^−^, *m*/*z* (ESI positive): 321.0 [M + H]^+^.

**7-((3-fluorobenzyl)oxy)-4-methylbenzo[e][1,2]oxathiine 2,2-dioxide** (**20**)

Compound **20** was obtained according to the general procedure reported earlier with 3-fluorobenzyl bromide as starting material. Yield 75%; m.p. 214–215 °C; silica gel TLC Rf 0.55 (EtOAc/Hexane 50% *v*/*v*): 7.74 (d, *J* = 9.0 Hz, 1H, Ar-*H*), 7.50 (m, 1H, Ar-*H*), 7.35 (m, 2H, Ar-*H*), 7.25 (m, 2H, Ar-*H*), 7.20 (d, *J* = 2.2 Hz, 1H, Ar-*H*), 7.13 (dd, *J* = 8.3 2.2 Hz, 1H, Ar-*H*), 5.30 (s, 2H, C*H*_2_), 2.37 (s, 3H, C*H*_3_); δ_H_ (400 MHz, DMSO-d_6_); δ_C_ (400 MHz, DMSO-d_6_): 164.5 (d, *J*^1^ = 168.2 Hz), 162.0, 152.4, 146.5, 140.1 (d, *J*^3^ = 7.9 Hz), 131.6 (d, *J*^3^ = 9.6 Hz), 129.4, 124.8 (d, *J*^4^ = 2.5 Hz), 117.4, 116.0 (d, *J*^2^ = 19.6 Hz), 115.6 (d, *J*^2^ = 20.5), 114.8, 114.3, 105.8, 70.2, 20.0; *m*/*z* (ESI negative): 318.9 [M − H]^−^, *m*/*z* (ESI positive): 321.0 [M + H]^+^.

**7-((4-nitrobenzyl)oxy)-4-methylbenzo[e][1,2]oxathiine 2,2-dioxide** (**21**)

Compound **21** was obtained according to the general procedure reported earlier with 4-nitrobenzyl bromide as starting material. Yield 64%; m.p. 247–249 °C; silica gel TLC Rf 0.44 (EtOAc/Hexane 50% *v*/*v*); δ_H_ (400 MHz, DMSO-d_6_): 8.27 (d, *J* = 8.9 Hz, 2H, Ar-*H*), 7.37 (d, *J* = 8.9 Hz, 2H, Ar-*H*), 7.70 (d, *J* = 8.4 Hz, 1H, Ar-*H*), 7.20 (s, 1H, Ar-*H*), 7.14 (d, *J* = 2.2 Hz, 1H, Ar-*H*), 7.10 (dd, *J* = 8.4 2.2 Hz,1H, Ar-*H*), 5.41 (s, 2H, C*H*_2_), 2.32 (s, 3H, C*H*_3_); δ_C_ (400 MHz, DMSO-d_6_): 161.8, 152.4, 148.2, 146.5, 145.0, 132.8, 129.5, 124.7, 117.5, 115.0, 114.3, 105.8, 69.8, 20.0; *m*/*z* (ESI negative): 345.9 [M − H]^−^, *m*/*z* (ESI positive): 348.0 [M + H]^+^.

**7-((3-nitrobenzyl)oxy)-4-methylbenzo[e][1,2]oxathiine 2,2-dioxide** (**22**)

Compound **22** was obtained according to the general procedure reported earlier with 3-nitrobenzyl bromide as starting material. Yield 89%; m.p. 239–241 °C; silica gel TLC Rf 0.57 (EtOAc/Hexane 50% *v*/*v*); δ_H_ (400 MHz, DMSO-d_6_): 8.34 (s, 1H, Ar-*H*), 8.21 (d, *J* = 8.1 Hz, 1H, Ar-*H*), 7.93 (d, *J* = 8.1 Hz,1H, Ar-*H*), 7.73 (dd, *J* = 8.1 Hz, 1H, Ar-*H*), 7.71 (d, *J* = 8.3 Hz, 1H, Ar-*H*), 7.20 (s, 1H, Ar-*H*), 7.19 (d, *J* = 2.1 Hz, 1H, Ar-*H*), 7.12 (dd, *J* = 8.3 2.1 Hz, 1H, Ar-*H*), 5.39 (s, 2H, C*H*_2_), 2.35 (s, 3H, C*H*_3_); δ_C_ (400 MHz, DMSO-d_6_): 161.9, 152.4, 148.9, 146.5, 139.5, 135.3, 131.2, 129.1, 124.1, 123.3, 117.5, 115.0, 114.3, 105.8, 69.7, 20.0; *m*/*z* (ESI negative): 345.9 [M − H]^−^, *m*/*z* (ESI positive): 348.0 [M + H]^+^.

**3-(benzyloxy)dibenzo[c,e][1,2]oxathiine 6,6-dioxide** (**27**)

Compound **27** was obtained according to the general procedure earlier reported with benzyl bromide as starting material. Yield 51%; m.p. 232–235 °C; silica gel TLC Rf 0.69 (EtOAc/Hexane 50% *v*/*v*); δ_H_ (400 MHz, DMSO-d_6_): 8.20 (dd, *J* = 6.6 Hz, 2H, Ar-*H*), 8.02 (d, *J* = 7.0 Hz, 1H, Ar-*H*), 7.90 (dd, *J* = 7.0 Hz, 1H, Ar-*H*), 7.67 (dd, *J* = 7.0 Hz, 1H, Ar-*H*), 7.49 (d, *J* = 6.6 Hz, 2H, Ar-*H*), 7.39 (m, 3H, Ar-*H*), 7.24 (s, 1H, Ar-*H*), 7.17 (d, *J* = 8.8 Hz, 1H, Ar-*H*), 5.39 (s, 2H, C*H*_2_); δ_C_ (400 MHz, DMSO-d_6_): 161.8, 151.1, 137.2, 135.7, 132.0, 131.1, 129.8, 129.5, 129.2, 129.0, 128.1, 126.1, 124.7, 115.4, 114.6, 106.7, 71.6; *m*/*z* (ESI negative): 336.9 [M − H]^−^, *m*/*z* (ESI positive): 339.0 [M + H]^+^.

**3-((4-fluorobenzyl)oxy)dibenzo[c,e][1,2]oxathiine 6,6-dioxide** (**28**)

Compound **28** was obtained according to the general procedure reported earlier with 4-fluorobenzyl bromide as starting material. Yield 49%; m.p. 268–269 °C; silica gel TLC Rf 0.81 (EtOAc/Hexane 50% *v*/*v*): 8.26 (m, 2H, Ar-*H*), 8.07 (d, *J* = 7.3 Hz, 1H, Ar-*H*), 7.96 (m, 1H, Ar-*H*), 7.72 (dd, *J* = 7.3 Hz, 1H, Ar-*H*), 7.60 (m, 2H, Ar-*H*), 7.30 (m, 2H, Ar-*H*), 7.27 (d, *J* = 2.3 Hz, 1H, Ar-*H*), 7.22 (dd, *J* = 8.4 2.3 Hz, 1H, Ar-*H*), 5.26 (s, 2H, C*H*_2_); δ_H_ (400 MHz, DMSO-d_6_); δ_C_ (400 MHz, DMSO-d_6_): 163.1 (d, *J*^1^ = 186.4 Hz), 161.6, 151.1, 135.7, 133.4 (d, *J*^4^ = 3.7 HZ), 132.0, 131.4 (d, *J*^3^ = 8.6 Hz), 131.1, 129.8, 182.1, 126.1, 124.7, 116.5 (d, *J*^2^ = 21.5 Hz), 115.4, 114.6, 106.7, 70.4; *m*/*z* (ESI negative): 354.9 [M − H]^−^, *m*/*z* (ESI positive): 357.1 [M + H]^+^.

**3-((3-fluorobenzyl)oxy)dibenzo[c,e][1,2]oxathiine 6,6-dioxide** (**29**)

Compound **29** was obtained according to the general procedure reported earlier with 3-fluorobenzyl bromide as starting material. Yield 76%; m.p. 249–252 °C; silica gel TLC Rf 0.77 (EtOAc/Hexane 50% *v*/*v*); δ_H_ (400 MHz, DMSO-d_6_): 8.27 (d, *J* = 2.5 Hz, 1H, Ar-*H*), 8.25 (d, *J* = 3.3 Hz, 1H, Ar-*H*), 8.07 (d, *J* = 7.7 Hz, 1H, Ar-*H*), 7.95 (dd, *J* = 7.7 H, 1H, Ar-*H*), 7.72 (dd, *J* = 7.7 Hz, 1H, Ar-*H*), 7.51 (q, *J* = 7.7 Hz, 1H, Ar-*H*), 7.38 (m, 2H, Ar-*H*), 7.30 (d, *J* = 2.5 Hz, 1H, Ar-*H*), 7.23 (m, 2H, Ar-*H*), 5.31 (s, 2H, C*H*_2_); δ_C_ (400 MHz, DMSO-d_6_): 164.5 (d, *J*^1^ = 243.9 Hz), 161.5, 151.2, 140.2 (d, *J*^3^ = 7.3 HZ), 135.7, 132.0, 131.6 (d, *J*^3^ = 8.6 Hz), 131.1, 129.9, 128.2, 126.2, 124.9 (d, *J*^4^ = 2.6 Hz), 124.7, 116.0 (d, *J*^2^ = 20.4 Hz), 115.7 (d, *J*^2^ = 21.3 Hz), 115.4, 114.8, 106.8, 70.2; *m*/*z* (ESI negative): 354.9 [M − H]^−^, *m*/*z* (ESI positive): 357.0 [M + H]^+^.

**3-((4-nitrobenzyl)oxy)dibenzo[c,e][1,2]oxathiine 6,6-dioxide** (**30**)

Compound **30** was obtained according to the general procedure reported earlier with 4-nitrobenzyl bromide as starting material. Yield 72%; m.p. 264–266 °C; silica gel TLC Rf 0.43 (EtOAc/Hexane 50% *v*/*v*); δ_H_ (400 MHz, DMSO-d_6_): 8.28 (d, *J* = 8.1 Hz, 2H, Ar-*H*), 8.23 (d, *J* = 8.8 Hz, 2H, Ar-*H*), 8.03 (d, *J* = 7.7 Hz, 1H, Ar-*H*), 7.91 (dd, *J* = 7.7 Hz, 1H, Ar-*H*), 7.76 (d, *J* = 8.1 H, 2H, Ar-*H*), 7.68 (dd, *J* = 7.7 Hz, 1H, Ar-*H*), 7.27 (s, 1H, Ar-*H*), 7.20 (d, *J* = 8.8 Hz, 1H, Ar-*H*), 5.42 (s, 2H, C*H*_2_); δ_C_ (400 MHz, DMSO-d_6_): 161.3, 151.1, 148.2, 145.1, 135.7, 131.9, 131.1, 130.0, 129.5, 128.3, 126.2, 124.8, 124.7, 115.4, 115.0, 106.8, 69.8; *m*/*z* (ESI negative): 382.0 [M − H]^−^, *m*/*z* (ESI positive): 383.9 [M + H]^+^.

**3-((3-nitrobenzyl)oxy)dibenzo[c,e][1,2]oxathiine 6,6-dioxide** (**31**)

Compound **31** was obtained according to the general procedure reported earlier with 3-nitrobenzyl bromide as the starting material. Yield 70%; m.p. 248–251 °C; silica gel TLC Rf 0.56 (EtOAc/Hexane 50% *v*/*v*): 8.36 (s, 1H, Ar-*H*), 8.23 (s, *J* = 8.6 Hz, 3H, Ar-*H*), 8.03 (d, *J* = 7.7 Hz, 1H, Ar-*H*), 7.96 (d, *J* = 7.7 Hz, 1H, Ar-*H*), 7.91 (dd, *J* = 7.7 Hz, 1H, Ar-*H*), 7.73 (dd, *J* = 7.7 Hz, 1H, Ar-*H*), 7.68 (dd, *J* = 7.7 Hz, 1H, Ar-*H*), 7.29 (d, *J* = 2.3 Hz, 1H, Ar-*H*), 7.22 (dd, *J* = 8.6 2.3 Hz, 1H, Ar-*H*), 5.41 (s, 2H, C*H*_2_); δ_H_ (400 MHz, DMSO-d_6_); δ_C_ (400 MHz, DMSO-d_6_): 161.3, 151.1, 148.9, 139.6, 135.7, 135.4, 131.9, 131.2, 131.1, 129.9, 128.3, 126.2, 124.7, 124.1, 123.4, 115.4, 114.9, 106.8, 69.7; *m*/*z* (ESI negative): 382.0 [M − H]^−^, *m*/*z* (ESI positive): 384.0 [M + H]^+^.

**General synthetic procedure for ethyl benzo[e][1,2]oxathiine-3-carboxylate 2,2-dioxide (34) and ethyl 7-methoxybenzo[e][1,2]oxathiine-3-carboxylate 2,2-dioxide (35)**

A solution of ethyl 2-(chlorosulfonyl)acetate (1.5 equiv.) in DCE (5 mL), prepared as reported by Liu et al.,^1^ was added dropwise in a sealed tube to a solution of **32** or **33** (1 g, 1 equiv.) and dry Py (2 equiv.) in DCE (3 mL). The resulting suspension was stirred at 90 °C for 4 h. Slush and HCl were added to the reaction mixture and the suspension was extracted in EtOAc (3 × 25 mL). The collected organic phase was washed with K_2_CO_3_ s.s. (3 × 20 mL), dried with Na_2_SO_4_, filtered and evaporated to give product **34** and **35** as pale yellow powders in good yield and high purity.

**ethyl benzo[e][1,2]oxathiine-3-carboxylate 2,2-dioxide (34)**

Compound **34** was obtained according to the general procedure reported earlier with salicyl aldehyde (**32**) as starting material. Yield 43%; m.p. 111–113 °C; silica gel TLC Rf 0.54 (EtOAc/Hexane 50% *v*/*v*); δ_H_ (400 MHz, DMSO-d_6_): 8.59 (s, 1H, Ar-*H*), 8.02 (d, *J* = 7.6 Hz, 1H, Ar-*H*), 7.79 (dd, *J* = 7.6 Hz, 1H, Ar-*H*), 7.59 (m, 2H, Ar-*H*), 4.43 (q, *J* = 6.5 Hz, 2H, C*H*_2_), 1.37 (dd, *J* = 6.5 Hz, 3H, C*H*_3_); δ_C_ (400 MHz, CDCl_3_-d_3_): 159.7, 152.4, 142.2, 131.0, 126.3, 118.9, 118.8, 63.1, 14.1.

**ethyl 7-methoxybenzo[e][1,2]oxathiine-3-carboxylate 2,2-dioxide (35)**

Compound **35** was obtained according to the general procedure reported earlier with 4-methoxysalicylaldehyde (**33**) as starting material. Yield 41%; m.p. 167–169 °C; silica gel TLC Rf 0.49 (EtOAc/Hexane 50% *v*/*v*); δ_H_ (400 MHz, DMSO-d_6_): 8.51 (s, 1H, Ar-*H*), 7.94 (d, *J* = 8.6 Hz, 1H, Ar-*H*), 7.22 (d, *J* = 2.2 Hz, 1H, Ar-*H*), 7.12 (dd, *J* = 8.6 2.2 Hz, 1H, Ar-*H*), 4.40 (q, *J* = 6.5 Hz, 2H, C*H*_2_), 3.95 (s, 3H, C*H*_3_), 1.35 (dd, *J* = 6.5 Hz, 3H, C*H*_3_); δ_C_ (400 MHz, CDCl_3_-d_3_): 164.8, 160.1, 154.4, 142.4, 132.4, 123.8, 113.4, 111.9, 103.8, 62.8, 56.1, 14.1.

**General synthetic procedure for benzo[e][1,2]oxathiine-3-carboxylic acid 2,2-dioxide (36) and 7-methoxybenzo[e][1,2]oxathiine-3-carboxylic acid 2,2-dioxide (37)**

NaOH 5M (7 equiv.) was added to a solution of appropriate ethyl esthers **34** or **35** (1g, 1 equiv.) in EtOH (20 mL), and the suspension was heated at reflux for 1h. The reaction mixuture was cooled and slushed, and HCl 6M were added to pH = 2. The resulting precipitate was filtered and washed with H_2_O to give us compounds **36** and **37** as a white powder in high yield and purity.

**benzo[e][1,2]oxathiine-3-carboxylic acid 2,2-dioxide (36)**

Compound **36** was obtained according to the general procedure reported earlier with **34** as starting material. Yield 81%; m.p. 105–107 °C; silica gel TLC Rf 0.04 (MeOH/DCM 5% *v*/*v*); δ_H_ (400 MHz, DMSO-d_6_): 14.57 (s, 1H, exchange with D_2_O, COO*H*), 8.50 (s, 1H, Ar-*H*), 7.98 (d, *J* = 7.4 Hz, 1H, Ar-*H*), 7.71 (dd, *J* = 7.4 Hz, 1H, Ar-*H*), 7.55 (m, 2H, Ar-*H*); δ_C_ (400 MHz, CDCl_3_-d_3_): 169.1, 159.4, 142.1, 132.5, 126.7, 118.8, 118.5.

**7-methoxybenzo[e][1,2]oxathiine-3-carboxylic acid 2,2-dioxide (37)**

Compound **37** was obtained according to the general procedure reported earlier with **35** as starting material. Yield 73%; m.p. 150–151 °C; silica gel TLC Rf 0.01 (MeOH/DCM 5% *v*/*v*); δ_H_ (400 MHz, DMSO-d_6_): 14.31 (s, 1H, exchange with D_2_O, COO*H*), 8.41 (s, 1H, Ar-*H*), 7.89 (d, *J* = 8 Hz, 1H, Ar-*H*), 7.18 (d, *J* = 2.1 Hz, 1H, Ar-*H*), 7.10 (dd, *J* = 8 2.1 Hz, 1H, Ar-*H*), 3.94 (s, 3H, C*H*_3_); δ_C_ (400 MHz, CDCl_3_-d_3_): 170.0, 157.6, 154.7, 140.6, 132.0, 123.1, 113.4, 111.8, 103.9, 56.0.

**General synthetic procedure for products 38–47**

PyBOP (1.2 equiv.) and appropriate aniline (1.2 equiv.) were added at 0 °C to a solution of **36** or **37** (0.2 g, 1 equiv.) in dry DMF (2 mL) under nitrogen atmosphere. DIPEA (3 equiv.) were added dropwise at 0 °C and the resulting solution was stirred o.n. at RT. The reaction mixture was quenched with slush and HCl 6M and the H_2_O-phase was extracted in EtOAc (3 × 30 mL). The collected organic phases were washed with HCl 1M (3 × 20 mL), NaHCO_3_ (2 × 20 mL) and brine (3 × 20 mL), then the organic phase was dried with Na_2_SO_4_, filtered and evaporated under vacuum to obtain. All the products were purified by silica gel chromathography (MeOH/DCM 0.5% *v*/*v*).

***N*-phenylbenzo[e][1,2]oxathiine-3-carboxamide 2,2-dioxide (38)**

Compound **38** was obtained according to the general procedure reported earlier with aniline as starting material. Yield 60%; m.p. 192–195 °C; silica gel TLC Rf 0.82 (MeOH/DCM 5% *v*/*v*); δ_H_ (400 MHz, DMSO-d_6_): 10.83 (s, 1H, exchange with D_2_O, CON*H*), 8.35 (s, 1H, Ar-*H*), 7.92 (d, *J* = 8.0 Hz, 1H, Ar-*H*), 7.74 (m, 3H, Ar-*H*), 7.58 (m, 2H, Ar-*H*), 7.44 (dd, *J* = 7.3 Hz, 2H, Ar-*H*), 7.21 (dd, *J* = 7.3 Hz, 1H, Ar-*H*); δ_C_ (400 MHz, DMSO-d_6_): 159.0, 152.1, 139.1, 138.5, 135.2, 132.4, 132.3, 130.0, 127.9, 125.6, 121.1, 120.0, 119.8; *m*/*z* (ESI negative): 299.9 [M − H]^−^, *m*/*z* (ESI positive): 302.0 [M + H]^+^.

***N*-(4-bromophenyl)benzo[e][1,2]oxathiine-3-carboxamide 2,2-dioxide (39)**

Compound **39** was obtained according to the general procedure reported earlier with 4-bromoaniline as starting material. Yield 21%; m.p. 249–252 °C; silica gel TLC Rf 0.88 (MeOH/DCM 5% *v*/*v*); δ_H_ (400 MHz, DMSO-d_6_): 10.96 (s, 1H, exchange with D_2_O, CON*H)*, 8.36 (s, 1H, Ar-*H*), 7.92 (dd, *J* = 7.7 1.4 Hz, 1H, Ar-*H*), 7.76 (ddd, *J* = 7.7 1.4 Hz, 1H, Ar-*H*), 7.69 (d, *J* = 8.8 Hz, 2H Ar-*H*), 7.62 (d, *J* = 8.8 Hz, 2H, Ar-*H*), 7.58 (m, 2H, Ar-*H*); δ_C_ (400 MHz, DMSO-d_6_): 159.1, 152.1, 138.9, 138.5, 135.4, 132.8, 132.3, 132.2, 128.0, 123.0, 119.9, 119.8, 117.4; *m*/*z* (ESI negative): 379.9 [M − H]^−^, *m*/*z* (ESI positive): 381.9 [M + H]^+^.

***N*-(3-bromophenyl)benzo[e][1,2]oxathiine-3-carboxamide 2,2-dioxide (40)**

Compound **40** was obtained according to the general procedure reported earlier with 3-bromoaniline as starting material. Yield 38%; m.p. 228–229 °C; silica gel TLC Rf 0.90 (MeOH/DCM 5% *v*/*v*); δ_H_ (400 MHz, DMSO-d_6_): 10.96 (s, 1H, exchange with D_2_O, CON*H)*, 8.38 (s, 1H, Ar-*H*), 8.01 (s, 1H, Ar-*H*), 7.91 (dd, *J* = 7.9 1.7 Hz, 1H Ar-*H*), 7.77 (ddd, *J* = 7.9 1.7 Hz, 1H, Ar-*H*), 7.67 (m, 1H, Ar-*H*), 7.58 (m, 2H, Ar-*H*), 7.41 (d, *J* = 5.5 Hz, 2H, Ar-*H*); δ_C_ (400 MHz, DMSO-d_6_): 159.2, 152.2, 140.7, 139.0, 135.4, 132.3, 132.1, 132.0, 128.2, 128.0, 123.5, 122.6, 119.9, 119.8, 118.2; *m*/*z* (ESI negative): 379.9 [M − H]^−^, *m*/*z* (ESI positive): 381.9 [M + H]^+^.

***N*-(4-methoxyphenyl)benzo[e][1,2]oxathiine-3-carboxamide 2,2-dioxide (41)**

Compound **41** was obtained according to the general procedure reported earlier with 4-methoxyaniline as starting material. Yield 37%; m.p. 231–233 °C; silica gel TLC Rf 0.82 (MeOH/DCM 5% *v*/*v*); δ_H_ (400 MHz, DMSO-d_6_): 10.70 (s, 1H, exchange with D_2_O, CON*H)*, 8.30 (s, 1H, Ar-*H*), 7.90 (dd, *J* = 7.6 1.8 Hz, 1H, Ar-*H*), 7.75 (ddd, *J* = 7.6 1.8 Hz, 1H, Ar-*H*), 7.63 (d, *J* = 9.0 Hz, 2H, Ar-*H*), 7.56 (m, 2H, Ar-*H*), 7.00 (d, *J* = 9.0 Hz, 2H, Ar-*H*), 3.80 (s, 3H, C*H*_3_); δ_C_ (400 MHz, DMSO-d_6_): 158.6, 157.1, 152.1, 138.1, 135.1, 132.6, 132.2, 132.1, 127.9, 122.6, 120.0, 119.8, 115.1, 56.3; *m*/*z* (ESI negative): 329.9 [M − H]^−^, *m*/*z* (ESI positive): 332.0 [M + H]^+^.

***N*-(3-methoxyphenyl)benzo[e][1,2]oxathiine-3-carboxamide 2,2-dioxide (42)**

Compound **42** was obtained according to the general procedure reported earlier with 3-methoxyaniline as starting material. Yield 31%; m.p. 219–221 °C; silica gel TLC Rf 0.94 (MeOH/DCM 5% *v*/*v*); δ_H_ (400 MHz, DMSO-d_6_): 10.79 (s, 1H, exchange with D_2_O, CON*H)*, 8.34 (s, 1H, Ar-*H*), 7.91 (dd, *J* = 7.5 1.3 Hz, 1H, Ar-*H*), 7.76 (ddd, *J* = 7.5 1.3 Hz, 1H, Ar-*H*), 7.57 (m, 2H, Ar-*H*), 7.33 (m, 3H, Ar-*H*), 6.79 (dd, *J* = 8.4 1.8 Hz, 1H, Ar-*H*), 3.81 (s, 3H, C*H*_3_); δ_C_ (400 MHz, DMSO-d_6_): 160.6, 159.0, 152.1, 140.3, 138.6, 135.3, 132.4, 132.3, 130.8, 128.0, 119.9, 119.8, 113.3, 111.1, 106.7, 56.1; *m*/*z* (ESI negative): 329.9 [M − H]^−^, *m*/*z* (ESI positive): 332.0 [M + H]^+^.

**7-methoxy-N-phenylbenzo[e][1,2]oxathiine-3-carboxamide 2,2-dioxide (43)**

Compound **43** was obtained according to the general procedure reported earlier with aniline as starting material. Yield 50%; m.p. 241–244 °C; silica gel TLC Rf 0.76 (MeOH/DCM 5% *v*/*v*); δ_H_ (400 MHz, DMSO-d_6_): 10.71 (s, 1H, exchange with D_2_O, CON*H)*, 8.29 (s, 1H, Ar-*H*), 7.83 (d, *J* = 8.4 Hz, 1H, Ar-*H*), 7.71 (d, *J* = 7.1 Hz, 2H, Ar-*H*), 7.43 (dd, *J* = 7.1 Hz, 2H, Ar-*H*), 7.17 (m, 3H, Ar-*H*), 3.94 (s, 3H, C*H*_3_); δ_C_ (400 MHz, DMSO-d_6_): 164.9, 159.3, 154.1, 139.3, 138.9, 133.6, 129.9, 129.0, 125.4, 121.0, 114.7, 112.7, 105.1, 57.4; *m*/*z* (ESI negative): 329.9 [M − H]^−^, *m*/*z* (ESI positive): 332.0 [M + H]^+^.

***N*-(4-bromophenyl)-7methoxybenzo[e][1,2]oxathiine-3-carboxamide 2,2-dioxide (44)**

Compound **44** was obtained according to the general procedure reported earlier with 4-bromoaniline as starting material. Yield 38%; m.p. 272–275 °C; silica gel TLC Rf 0.92 (MeOH/DCM 5% *v*/*v*); δ_H_ (400 MHz, DMSO-d_6_): 10.83 (s, 1H, exchange with D_2_O, CON*H)*, 8.31 (s, 1H, Ar-*H*), 7.83 (d, *J* = 8.4 Hz, 1H, Ar-*H*), 7.68 (d, *J* = 9.1 Hz, 2H, Ar-*H*), 7.61 (d, *J* = 9.1 Hz, 2H, Ar-*H*), 7.22 (d, *J* = 2.2 Hz, 1H, Ar-*H*), 7.12 (dd, *J* = 8.4 2.2 Hz, 1H, Ar-*H*), 3.94 (s, 3H, C*H*_3_); δ_C_ (400 MHz, DMSO-d_6_): 165.0, 159.4, 154.1, 139.2, 138.7, 133.7, 132.8, 128.8, 122.9, 117.2, 114.8, 112.7, 105.2, 57.4; *m*/*z* (ESI negative): 407.9 [M − H]^−^, *m*/*z* (ESI positive): 411.9 [M + H]^+^.

***N*-(3-bromophenyl)-7-methoxybenzo[e][1,2]oxathiine-3-carboxamide 2,2-dioxide (45)**

Compound **45** was obtained according to the general procedure reported earlier with 3-bromoaniline as starting material. Yield 32%; m.p. 218–220 °C; silica gel TLC Rf 0.87 (MeOH/DCM 5% *v*/*v*); δ_H_ (400 MHz, DMSO-d_6_): 10.80 (s, 1H, exchange with D_2_O, CON*H)*, 8.26 (s, 1H, Ar-*H*), 7.96 (s, 1H, Ar-*H*), 7.78 (d, *J* = 8.3 Hz, 1H, Ar-*H*), 7.62 (d, *J* = 4.5 Hz, 1H, Ar-*H*), 7.35 (m, 2H, Ar-*H*), 7.18 (d, *J* = 2.2 Hz, 1H, Ar-*H*), 7.08 (dd, *J* = 8.3 2.2 Hz, 1H, Ar-*H*), 3.90 (s, 3H, C*H*_3_); δ_C_ (400 MHz, DMSO-d_6_): 165.1, 159.6, 154.1, 140.8, 139.4, 133.7, 132.0, 128.6, 128.0, 123.4, 122.6, 119.8, 114.8, 112.6, 105.2, 57.4; *m*/*z* (ESI negative): 409.9 [M − H]^−^, *m*/*z* (ESI positive): 411.9 [M + H]^+^.

***N*-(4-methoxyphenyl)-7-methoxybenzo[e][1,2]oxathiine-3-carboxamide 2,2-dioxide (46)**

Compound **46** was obtained according to the general procedure reported earlier with 4-methoxyaniline as starting material. Yield 58%; m.p. 238–240 °C; silica gel TLC Rf 0.88 (MeOH/DCM 5% *v*/*v*): 10.57 (s, 1H, exchange with D_2_O, CON*H)*, 8.24 (s, 1H, Ar-*H*), 7.82 (d, *J* = 8.2 Hz, 1H, Ar-*H*), 7.62 (d, *J* = 8.8 Hz, 2H, Ar-*H*), 7.21 (d, *J* = 2.2 Hz, 1H, Ar-*H*), 7.12 (dd, *J* = 8.2 2.2 Hz, 1H, Ar-*H*), 6.99 (d, *J* = 8.8 Hz, 2H, Ar-*H*), 3.97 (s, 3H, C*H*_3_), 3.82 (s, 3H, C*H*_3_); δ_H_ (400 MHz, DMSO-d_6_); δ_C_ (400 MHz, DMSO-d_6_): 164.8, 158.9, 157.1, 154.0, 138.4, 133.5, 132.2, 129.2, 122.6, 115.1, 114.7, 112.8, 105.1, 57.4, 56.3; *m*/*z* (ESI negative): 359.9 [M − H]^−^, *m*/*z* (ESI positive): 362.0 [M + H]^+^.

***N*-(3-methoxyphenyl)-7-methoxybenzo[e][1,2]oxathiine-3-carboxamide 2,2-dioxide (47)**

Compound **47** was obtained according to the general procedure reported earlier with 3-methoxyaniline as starting material. Yield 41%; m.p. 222–225 °C; silica gel TLC Rf 0.79 (MeOH/DCM 5% *v*/*v*); δ_H_ (400 MHz, DMSO-d_6_): 10.69 (s, 1H, exchange with D_2_O, CON*H)*, 8.27 (s, 1H, Ar-*H*), 7.37 (dd, *J* = 2.0 Hz, 1H, Ar-*H*), 7.32 (dd, *J* = 8.0 Hz, 1H, Ar-*H*), 7.27 (d, *J* = 8.0 Hz, 1H, Ar-*H*), 7.22 (d, *J* = 2.1 Hz, 1H, Ar-*H*), 7.12 (dd, *J* = 8.6 2.1 Hz, 1H, Ar-*H*), 6.77 (dd, *J* = 8.0 2.0 Hz, 1H, Ar-*H*), 3.94 (s, 3H, C*H*_3_), 3.80 (s, 3H, C*H*_3_); δ_C_ (400 MHz, DMSO-d_6_): 164.9, 160.6, 159.3, 154.1, 144.6, 140.4, 138.9, 133.6, 130.7, 129.0, 114.7, 113.2, 111.0, 106.7, 105.1, 57.4, 56.1; *m*/*z* (ESI negative): 359.9 [M − H]^−^, *m*/*z* (ESI positive): 362.0 [M + H]^+^.

### 3.2. Carbonic Anhydrase Inhibition

An Applied Photophysics stopped-flow instrument was used for assaying the CA catalyzed CO_2_ hydration activity [44]. Phenol red (at a concentration of 0.2 mM) has been used as indicator, working at the absorbance maximum of 557 nm, with 20 mM Hepes (pH 7.5) as buffer, and 20 mM Na_2_SO_4_ (for maintaining constant the ionic strength), following the initial rates of the CA-catalyzed CO_2_ hydration reaction for a period of 10–100 s. The CO_2_ concentrations ranged from 1.7 to 17 mM for the determination of the kinetic parameters and inhibition constants. For each inhibitor, at least six traces of the initial 5–10% of the reaction were used for determining the initial velocity. The uncatalyzed rates were determined in the same manner and subtracted from the total observed rates. Stock solutions of inhibitor (0.1 mM) were prepared in distilled–deionized water and dilutions up to 0.01 nM were done thereafter with the assay buffer. Inhibitor and enzyme solutions were preincubated together for 6h at room temperature prior to assay, in order to allow for the formation of the E-I complex. The inhibition constants were obtained by non-linear least-squares methods using PRISM 3 and the Cheng–Prusoff equation and represent the mean from at least three different determinations. The enzyme concentrations were in the range 5–16 nM. All CA isoforms were recombinant ones obtained in-house [47,48].

## 4. Conclusions

We report here a series of 7-substituted and 3-substituted sulfocoumarins, obtained by cyclization of 2-hydroxy-4-methoxybenzaldehyde (7), 2′-hydroxy-4′-methoxyacetophenon (8), 2-bromobenzenesulfonyl chloride (23), salicylaldehyde (32) or 4-methoxysalicylaldehyde (33) and possessing different substituents in position 3 or 7 of the heterocyclic ring. 7-Substituted sulfocoumarins resulted in being good inhibitors of hCA IX and XII, whilst 3-substituted sulfocoumarins showed a worsening in terms of inhibition potency on this isoform. A common feature of all the synthesized products was the absence of inhibition on hCA I and II. The most potent products belong to Type 1 group, probably due to the absence of the bulky group on the heterocyclic ring; particularly, sulfocoumarin 15 showed a nanomolar inhibition on hCA IX and XII and turned out to be the most potent inhibitor achieved (hCA IX: K_I_ = 22.9 nM; hCA XII: K_I_ = 19.2 nM). The structure-activity relationship for this class of CAIs has been expanded considering the synthesis of 3-substituted sulfocoumarines for the first time. The observed isoform-selective inhibition displayed here may be considered to be of interest for various biomedical applications.

## Figures and Tables

**Table 1:** Inhibition data of hCA isoforms I, II, IX and XII with sulfocoumarins **13**–**22**, **27**–**31**, **38**–**47** and the standard sulfonamide inhibitor AAZ by a stopped-flow CO_2_ hydrase assay [44].

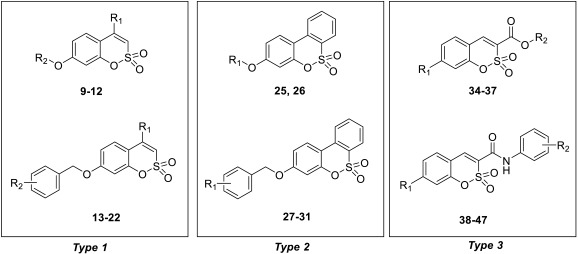						
Cmpd	K_I_ (nM) ^a,b^					
	R_1_	R_2_	CA I	CA II	CA IX	CA XII
**9**	H	OCH_3_	>100,000	15,360	74.9	61.4
**10**	CH_3_	OCH_3_	>100,000	8814	97.2	73.9
**11**	H	H	16,538	2896	55.8	42.5
**12**	CH_3_	H	9961	3857	81.7	55.0
**13**	H	H	>100,000	>100,000	55.6	28.3
**14**	H	4-F	>100,000	>100,000	22.9	19.2
**15**	H	3-F	>100,000	>100,000	28.4	46.0
**16**	H	4-NO_2_	>100,000	>100,000	69.8	94.7
**17**	H	3-NO_2_	>100,000	>100,000	40.2	77.5
**18**	CH_3_	H	>100,000	>100,000	49.8	8.6
**19**	CH_3_	4-F	>100,000	>100,000	14.3	41.7
**20**	CH_3_	3-F	>100,000	>100,000	33.2	25.7
**21**	CH_3_	4-NO_2_	>100,000	>100,000	89.7	100.8
**22**	CH_3_	3-NO_2_	>100,000	>100,000	61.4	136.0
**25**	OCH_3_	-	>100,000	>100,000	1286	965.8
**26**	H	-	42650	8997	558.9	414.2
**27**	H	-	>100,000	>100,000	680.5	910.7
**28**	4-F	-	>100,000	>100,000	754.9	653.8
**29**	3-F	-	>100,000	>100,000	615.1	566.4
**30**	4-NO_2_	-	>100,000	>100,000	964.7	1056
**31**	3-NO_2_	-	>100,000	>100,000	898.1	1369
**34**	H	CH_2_CH_3_	>100,000	>100,000	1631	2594
**35**	OCH_3_	CH_2_CH_3_	>100,000	>100,000	1085	1528
**36**	H	H	4625	996.5	499.7	563.7
**37**	OCH_3_	H	7139	728.1	853.7	408.2
**38**	H	H	>100,000	>100,000	3288	2301
**39**	H	4-Br	>100,000	>100,000	34,350	58,980
**40**	H	3-Br	>100,000	>100,000	40,840	78,320
**41**	H	4-OCH_3_	>100,000	>100,000	8231	8550
**42**	H	3-OCH_3_	>100,000	>100,000	5424	3380
**43**	OCH_3_	H	>100,000	>100,000	2429	1594
**44**	OCH_3_	4-Br	>100,000	>100,000	51,460	42,520
**45**	OCH_3_	3-Br	>100,000	>100,000	64,380	76,540
**46**	OCH_3_	4-OCH_3_	>100,000	>100,000	4367	6547
**47**	CH_3_	3-OCH_3_	>100,000	>100,000	6558	2469
**AAZ**	-	-	250	12.0	25.0	5.7

^a^. Mean from 3 different assays by a stopped flow technique (errors in the range of 5−10% of the reported values); ^b^. 6 h incubation.

## Data Availability

Compounds or related data available directly from the authors.
